# Measuring emergency department crowding: comparing the NEDOCS and Fenice crowding scores against staff perception

**DOI:** 10.1007/s11739-025-04216-7

**Published:** 2025-12-12

**Authors:** Riccardo Boverio, Fabiola Signorini, Carlotta Rossi, Roberta Irene Maria Bellini, Cristian Zanelli, Giulia Irene Ghilardi, Daniela Zatelli, Giovanni Nattino, Guido Bertolini

**Affiliations:** 1 Department of Emergency Medicine, Azienda Ospedaliero-Universitaria SS. Antonio e Biagio e Cesare Arrigo, Alessandria, Italy; 2 Department of Medical Epidemiology, Istituto di Ricerche Farmacologiche Mario Negri IRCCS, Via G. B. Camozzi 3, 24020 Ranica, (BG) Italy; 3 Strategy Deployment and Organisational Improvement Area, Azienda Ospedaliero-Universitaria SS. Antonio e Biagio e Cesare Arrigo, Alessandria, Italy

**Keywords:** Emergency medicine, Emergency department, Crowding, NEDOCS, Fenice crowding score, Healthcare staff perception

## Abstract

This study assesses the feasibility of collecting real-time perceptions of emergency department (ED) crowding from different professional roles and compares the recorded perceptions with two crowding indicators: the NEDOCS and the recently proposed Fenice score. We conducted a prospective observational study in an Italian university hospital. ED staff received two SMS questionnaires daily, assessing perceived crowding and workload pressure on 0–10 scales. Simultaneously, the NEDOCS and Fenice scores were recorded every 15 min. Spearman correlations, regression models, and generalized additive models were used to assess associations. Among 49 staff members, 830 valid on-shift responses were collected (response rate: 62.6%). Perceived crowding was generally high (median: 7; *Q*1–*Q*3: 5–9) and varied significantly by role (*p*-value < 0.001), with healthcare assistants and boarding nurses perceiving the ED as more crowded and process management nurses perceiving it as less crowded. The Fenice and NEDOCS scores correlated strongly with perceived crowding (*ρ*: 0.71 and 0.69; *p* value = 0.06) and moderately with workload pressure (*ρ* < 0.60). The Fenice score explained slightly more variance in perceived crowding (*R*^2^: 0.65 vs. 0.59). While the correlation between the two scores in the study ED was high (*ρ*: 0.85), a wide range of correlation coefficients emerged in a cohort of 83 Italian EDs (average *ρ*: 0.80; min–max: 0.24–0.99; *Q*1–*Q*3: 0.73–0.93). Real-time monitoring of ED staff perceptions is feasible and informative. Both the NEDOCS and Fenice scores correlate well with staff perception, but the simplicity and fully automatable implementation of the Fenice score make it a promising alternative to be evaluated in future studies.

**Supplementary Information:**

The online version contains supplementary material available at https://doi.org/10.1007/s11739-025-04216-7.

## Introduction

Despite the lack of a universally recognized definition [1], Emergency Departments (ED) crowding is a challenge for healthcare systems worldwide. It is caused by an imbalance between demand and available resources, whether structural, instrumental, or human [2]. The serious consequences of ED crowding are well established. Studies have shown that it places a significant burden on patients, including increased mortality [3], longer admission times [2, 4], ED length of stay [5], and longer hospitalizations. For healthcare staff, it has been associated with an increased risk of inappropriate care, a higher incidence of conflict and aggression, and an increased stress and burnout [6–8]. At the system level, it contributes to longer average hospitalization durations and increased use of diagnostic tests and therapeutic interventions. Therefore, accurately measuring crowding is essential.

The National Emergency Department Overcrowding Scale (NEDOCS) [9] is currently the most widely used tool in Italy to assess ED crowding levels, and one of the most studied measure of crowding perception worldwide [10]. The score is based on multiple factors that reflect the size of the healthcare facility (number of hospital and ED beds), the number and type of patients in the ED (total ED patients, number of ventilated patients, number of patients awaiting admission) and waiting times (maximum waiting time for medical visit and hospital admission).

However, despite its widespread use, the NEDOCS has two relevant limitations. First, it was developed on data collected in 2002 from eight medium-to-large university hospitals in the United States. As a result, the score cannot be assumed to accurately reflect current crowding conditions in settings that differ significantly from the original context, such as contemporary Italian EDs. Second, albeit the usefulness of the score relies on its automatic calculation using the data routinely recorded in clinical records, some of the necessary variables are difficult to extract, such as the number of ventilated patients or the number of patients awaiting hospital admission. Retrieving the former variable in real-time is challenging as the use of ventilators is often not integrated in the ED electronic health records (EHRs). Similarly, the latter variable relies on the availability of the time at which the physician in charge decides that the ED visit is complete and the patient requires hospitalization, which is rarely stored systematically in EHRs. These challenges have resulted in inconsistent approximations of the score’s components, which compromises the comparability of results across hospitals. Thus, the documented mismatch between NEDOCS values and the perception of the ED staff on crowding is not surprising [11].

Recently, the Fenice collaborative group (the Italian Group for Clinical Research in Emergency Medicine) [12] proposed a new crowding indicator [13], relying on one of the objective consequences of ED overcrowding: the waiting time for patients triaged with minor or deferrable urgency. The study also demonstrated the poor sensitivity of the NEDOCS on this specific outcome on a large cohort of all general EDs of the Lombardy region of Italy. The Fenice ED-crowding score is extremely simple to calculate and can be fully automated using routinely collected data, as, once the center-specific relationship is estimated on administrative data, it only requires the total number of patients present in the ED for real-time calculation.

The primary aim of this study was to evaluate the feasibility of collecting data to assess ED staff perceptions of crowding in Italian EDs. The secondary aim was assessing the level of agreement between the Fenice crowding score and staff perceptions and comparing this agreement with the one between NEDOCS and staff perceptions.

## Methods

### Study design and data collection

The study adopted a prospective observational design with repeated cross-sectional assessments. It was conducted in the general ED of the Santi Antonio e Biagio hospital in Alessandria (Italy), a large university hospital. The study ED approximately counts 45,000 adult visits annually. Between December 20, 2024 and February 20, 2025, the participating members of the ED staff received two daily SMS text messages containing a link to a questionnaire. Messages were sent at 10 AM and 4 PM using the system in place to record the satisfaction of ED patients after discharge. After specifying the professional role in the ED, on-shift operators were asked two questions: “What is the current level of overcrowding of the ED?” and “How rushed do you feel?” Both questions were rated on a scale ranging from 0 (not at all) to 10 (extremely). The healthcare staff involved in the study included the ED director, the chief nurse, physicians, healthcare assistants and nurses, for whom we recorded the specific role held when filling out the questionnaire (i.e., triage nurse, emergency room nurse, nurse in charge of boarding patients, and process management nurse).

Simultaneously, the Fenice and NEDOCS scores were computed and recorded with a 15-min frequency over the study period, as part of the hospital practice in place to measure ED crowding. Importantly, the participating members of the ED staff responded to the questionnaire blinded to the values of the two scores. Both crowding indicators are quantitative variables, with higher values corresponding to increased levels of crowding. In particular, the Fenice crowding indicator has a clear interpretation, as is expressed as the estimated average waiting time (in minutes) for the patients arriving to the ED with deferrable urgency. Details on the calculation of the two indicators are reported in the Supplementary Materials (Section S1).

To contextualize the findings of our monocentric study in a broad range of EDs with different characteristics, such as arrival volume, hospital size, and urban/rural location, the agreement between the NEDOCS and Fenice scores was also assessed in 83 general EDs in the Lombardy Region of Italy in the study period (Supplementary methods S1.1). The two scores were extracted from the database centralizing data from all EDs in the Region, which was leveraged to develop the Fenice crowding score [13].

This study relied solely on anonymous data. Healthcare staff voluntarily consented to complete the anonymous questionnaire about crowding perception. The Fenice and NEDOCS scores are computed on aggregate, and thus anonymous, data. Therefore, this study did not require approval from an Ethics Committee.

### Statistical analysis

One-way ANOVA was applied to test for differences in responses between roles, separately for the two questions. Furthermore, separately for each role, we used paired *t* tests to assess whether operators provided similar values for the perceived level of crowding and the feeling of being rushed.

Spearman correlation coefficient (*ρ*) was used to assess the overall association between each crowding indicator and staff perception of crowding. The two coefficients were compared by a standard Williams’ *t* test for overlapping correlations based on dependent groups [14]. Spearman’s *ρ* was also used to measure the association between the Fenice crowding score and NEDOCS levels in the study ED.

To assess the ability of the Fenice crowding score and NEDOCS to explain the perception of crowding while accounting for a possible heterogeneous association across professional roles, we used linear regression models. We considered two sets of models, one for each crowding indicator. Specifically, we separately regressed the response to questions (dependent variables) on the crowding indicator and its interaction with the professional role (independent variables). The ability of the crowding indicators to explain the staff perception was quantified with the determination coefficients (*R*^2^) of the models.

To study the shape of the relationship between the Fenice score and staff perception, we separately fit a Generalized Additive Model (GAM) for each staff role [15]. GAMs can be used to flexibly model the relationship between predictors and response with smooth functions, which were represented with thin plate regression splines [16].

To contextualize the association between the NEDOCS and Fenice scores observed in the study ED, we computed the correlation between the two scores in all 83 general EDs in Lombardy using the same metric (Spearman’s *ρ*).

Analyses were performed with R, version 4.2.1.

## Results

**Fig. 1:**
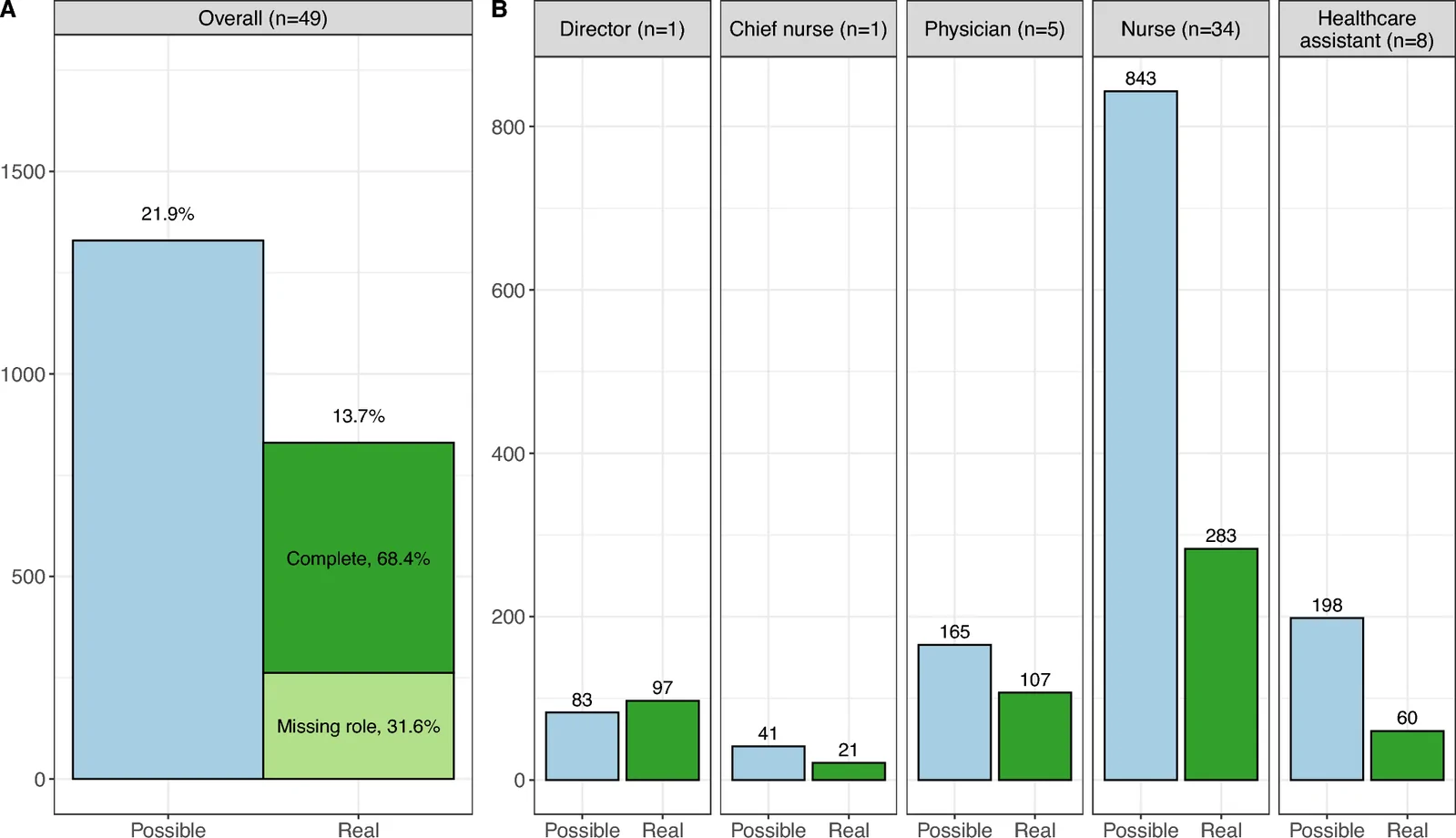
Comparison of the overall number of received answers with the possible number of responses, overall (panel **A**) and stratified by role (panel **B**)

A total of 49 healthcare professionals were involved in the study and reached by a total of 6047 SMS messages over the 2-month period. In addition to the ED director and the chief nurse, 5 ED physicians, 34 nurses and 8 healthcare assistants participated in the study. Figure 1 shows the proportion of responses overall (Panel A) and by role (Panel B), as well as the proportion of responses expected based on the number and duration of shifts per role. Of all the messages sent, 1329 (21.9%) were expected to reach the staff while on duty, whereas 830 (13.7%) responses were received. Of these, 262 responses (31.6%) were missing the professional role and were therefore excluded from the analyses stratified by role. The response rate was heterogeneous across professional figures. While, on average, the chief nurse responded to 21 out of the 41 messages received while on duty (51.2%), healthcare assistants responded to 30.3% of the messages (60 out of 198). Curiously, the response rate of the ED director was higher than 100%, as we recorded 97 responses out of the 83 messages sent while expected to be on duty. This is due to off-hour work in the ED. Overall, participation was almost constant over time, with a small decline over the second half of the study (Supplementary Figure S1). The vast majority of responses were recorded within 1 h of the message being sent (Supplementary Figure S2).

**Fig. 2:**
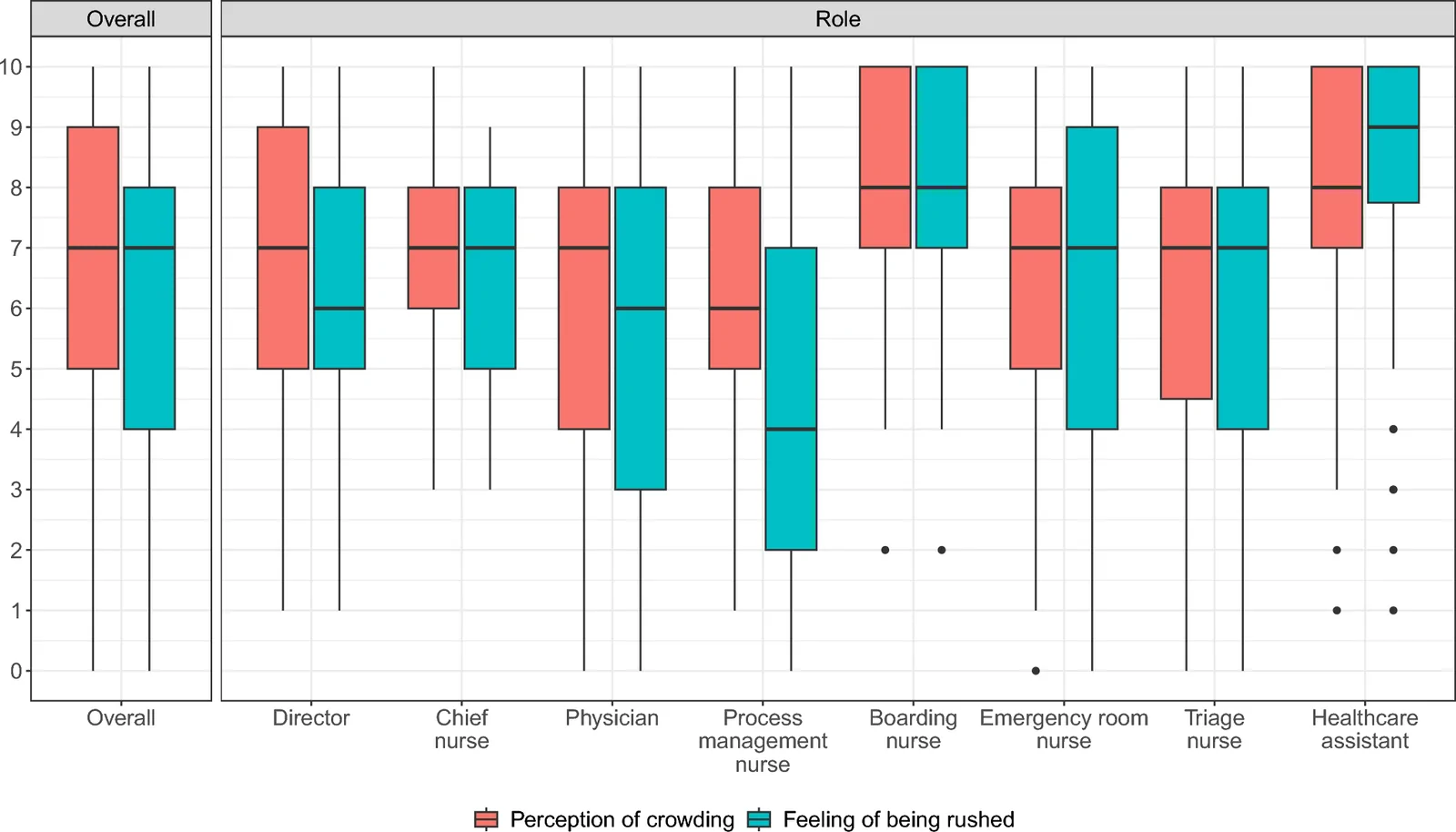
Distribution of the perception of crowding (question 1) and the feeling of being rushed (question 2), stratified by role

Figure 2 shows the distribution of the perceived level of crowding and the feeling of rush overall and by role. During the study period, the ED staff rarely perceived the ED as not crowded, as 75% of the values were greater than or equal to 5 (median: 7; *Q*1–*Q*3: 5–9). Significant heterogeneity emerged across roles (*p* value < 0.001). Healthcare assistants and boarding nurses perceived the ED as slightly more crowded than their colleagues did, whereas process management nurses perceived it as slightly less crowded. When considering the reported feeling of being rushed, we observed the same median value but lower *Q*1 and *Q*3 compared to the perception of crowding (median: 7; *Q*1–*Q*3: 4–8), and a high degree of heterogeneity across roles (*p* value < 0.001). Interestingly, we observed higher median values for the feeling of rush compared to the perception of crowding for healthcare assistants even though this difference was not statistically significant (*p* value = 0.24). In contrast, the feeling of rush was lower than the perception of overall crowding for the director (*p* value < 0.001), physicians (*p* value < 0.001), process management nurses (*p* value < 0.001) and, marginally, for triage nurses (*p* value = 0.046). We did not observe significant differences for the other roles (*p* values for chief nurse: 0.50; boarding nurse: 0.30; emergency nurse: 0.39).

**Fig. 3:**
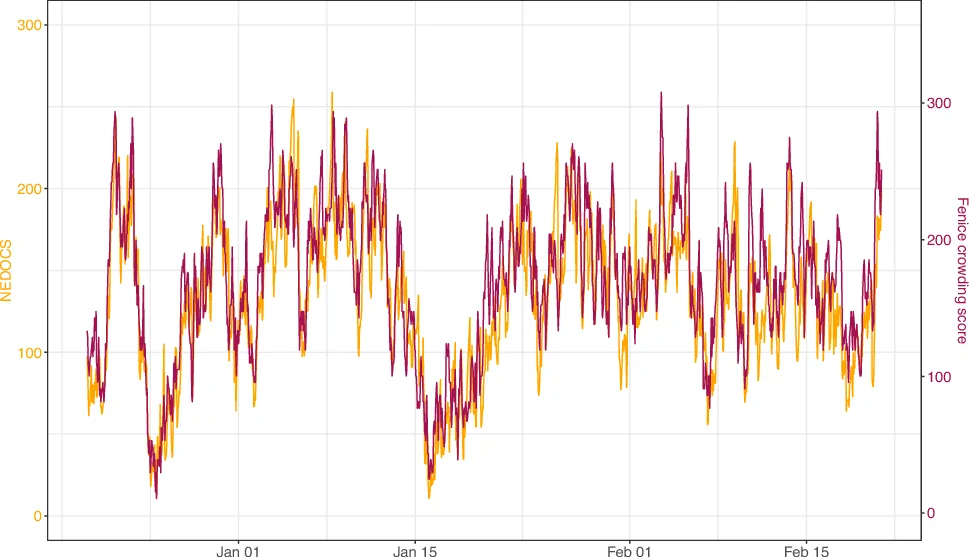
NEDOCS and Fenice crowding score hourly values during the study period in the studied ED

The hourly values of the NEDOCS (orange line) and Fenice crowding score (red line) over the 2-month study period are presented in Fig. 3. Although recorded on different scales, the two scores were strongly associated, as suggested by the very high correlation coefficient between them (*ρ* = 0.848; 95% CI 0.830–0.865). Thus, unsurprisingly, both the Fenice score and the NEDOCS were strongly associated with the staff perception of crowding, as documented by the correlation coefficients of 0.709 (95% CI 0.669–0.745) and 0.689 (95% CI 0.647–0.724) for the two scores, respectively. The difference between the two correlation coefficients was marginally non-significant (*p* value = 0.060). In contrast, when considering the feeling of being rushed, the associations were only moderate, as the correlation coefficients were 0.589 (95% CI 0.540–0.634) and 0.585 (95% CI 0.537–0.629) for the Fenice crowding score and NEDOCS, respectively (test comparing the coefficients: *p* value = 0.74). The linear model evaluating the ability of the Fenice score to explain staff perceptions of crowding while accounting for differences across roles attained an *R*^2^ of 0.65 (95% CI 0.61–0.70). The same value was 0.59 (95% CI 0.54–0.64) for the NEDOCS. The *R*^2^ values attained by the models explaining the feeling of being rushed were lower and, specifically, 0.54 (95% CI 0.49–0.59) for the Fenice crowding score and 0.51 (95% CI 0.45–0.57) for the NEDOCS.

**Fig. 4:**
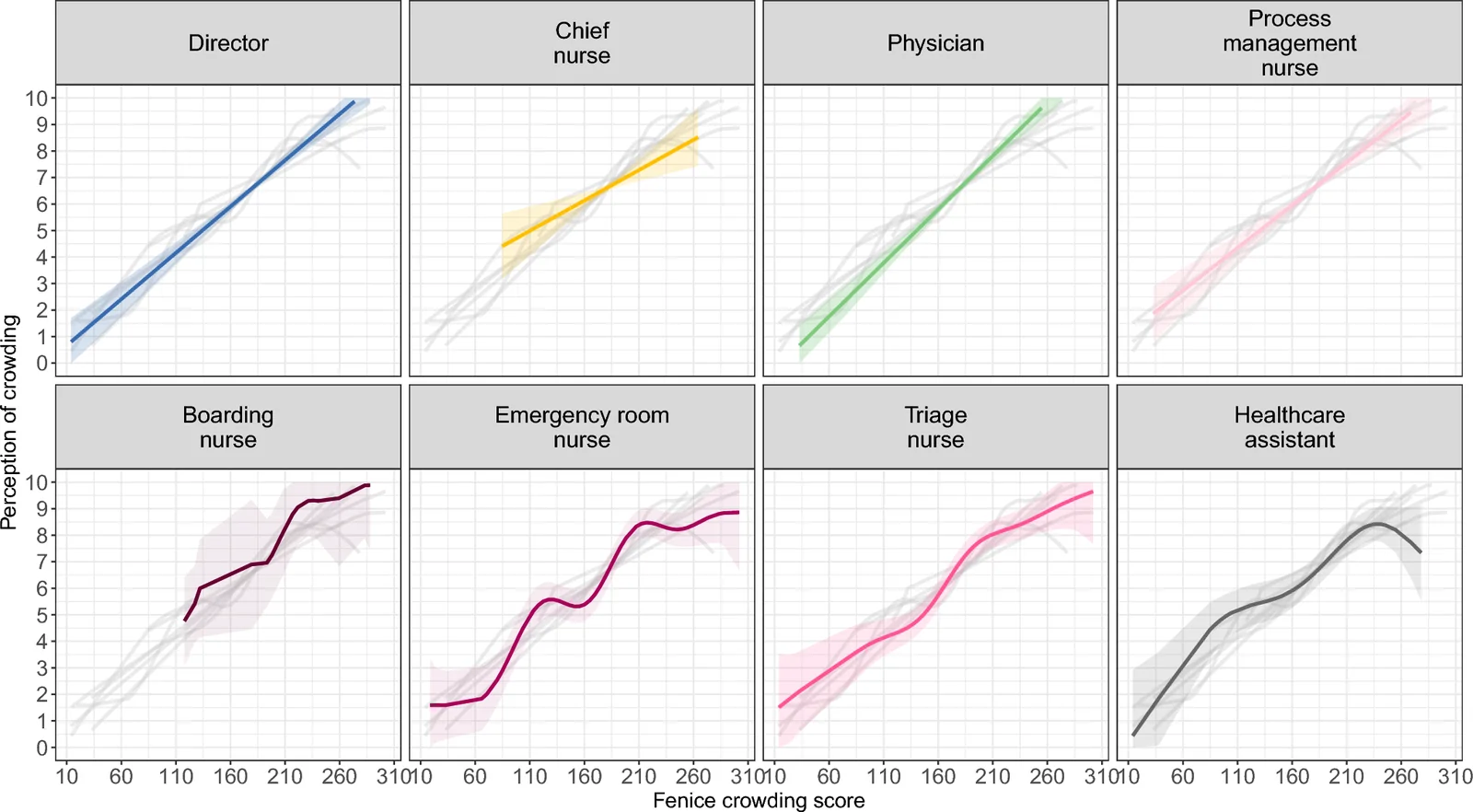
Estimated relationship between the Fenice score level and staff perception of crowding by role

The relationship between the Fenice crowding score level and the staff perception for the different roles, estimated with GAM models, is presented in Fig. 4. The resulting curves largely overlapped, confirming a similar and markedly positive relationship for all the different roles. Notably, while all trends were approximately linear, the relationship observed for the director, chief nurse, physicians, and process management nurses was extremely close to a straight line. In contrast, other roles tended toward a stepwise pattern. Specifically, for some members of the ED staff (particularly the emergency room nurses), the perception of crowding may not increase gradually with rising crowding levels but, rather, may intensify sharply when reaching certain threshold values.

**Fig. 5:**
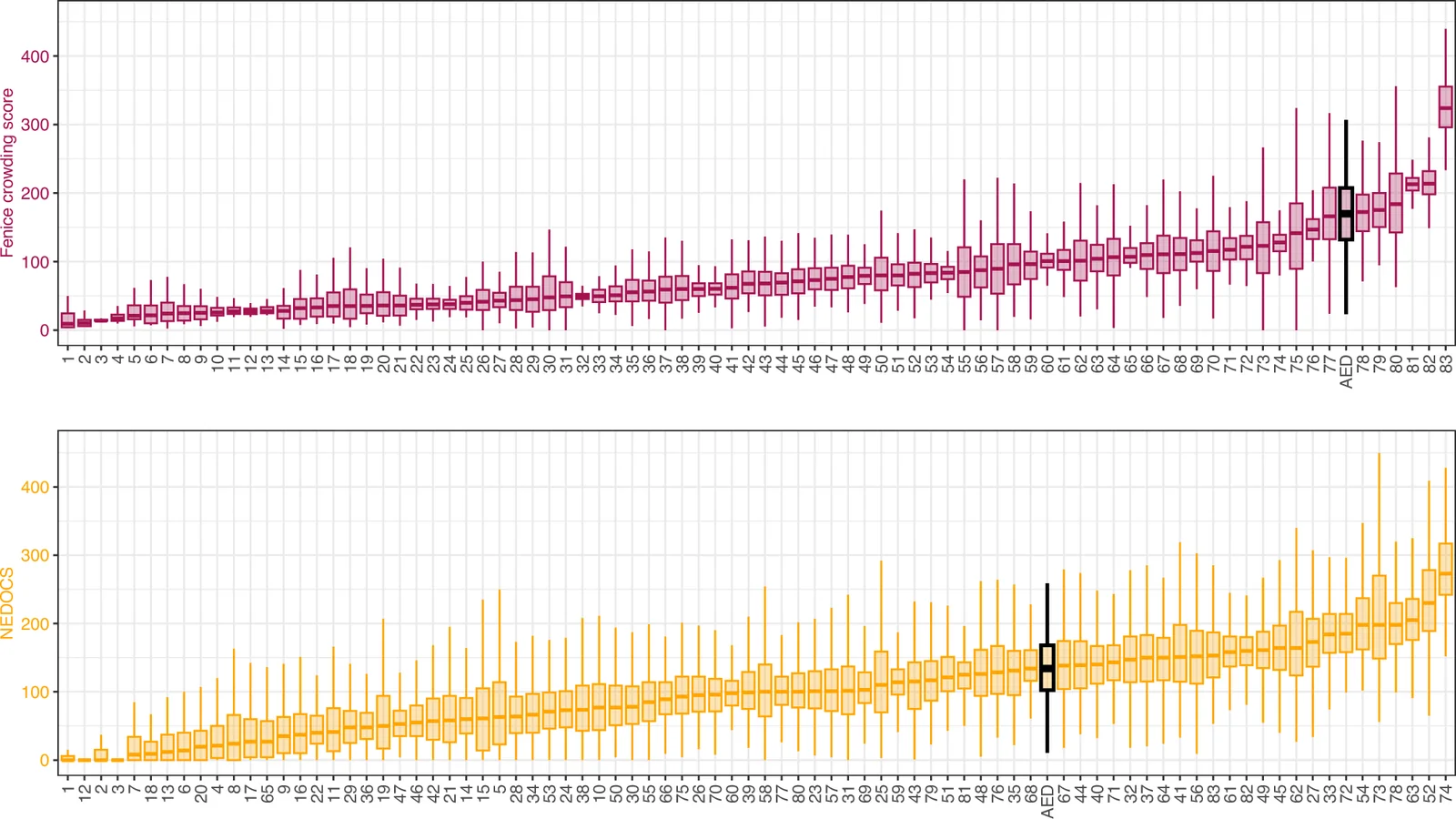
Distribution of the NEDOCS and Fenice crowding score during the study period in the studied ED (black) and in the 83 Lombardy EDs

To contextualize the strong association between NEDOCS and Fenice scores found in the Alessandria ED, the correlation between scores was computed for all Lombardy EDs. The correlation observed in the study center was not representative of those measured in the 83 EDs included in our database, which ranged from 0.238 to 0.992 (*Q*1–*Q*3: 0.728–0.934). In particular, 51 centers (61.4%) presented values lower than what observed in the study ED (Supplementary Figure S3). Figure 5 compares the distribution of the two indicators over the study period for the study ED (in black) and for the Lombardy EDs, sorted by the median value of each indicator. According to the Fenice crowding score, only six Lombardy EDs were, on average, more crowded than Alessandria’s ED. However, its rank changes when considering the NEDOCS score, with 24 EDs being more crowded than the study ED, based on this indicator. Similarly, many other changes in the ranking of the Lombardy EDs emerge. For example, the 27th and 33rd EDs according to the Fenice crowding score are among the ten most crowded centers when ranked by the NEDOCS, whereas the ED ranked 65th drops to the 13th position for the latter indicator.

## Discussion

This study provides initial evidence of the feasibility and value of measuring staff perceptions of ED crowding in real time, and of assessing the degree of agreement between these perceptions and two indicators of crowding: the widely used NEDOCS and the recently proposed Fenice crowding score.

Unfortunately, no gold standard measure of ED crowding currently exists. We decided to rely on the staff perception of crowding to evaluate the NEDOCS and Fenice scores because, despite its subjective nature, it remains the only indicator that directly targets the measurement of ED crowding. This is the rationale that drove the development of multiple crowding indicators, including the NEDOCS itself, which were grounded on staff perception measures. Albeit the Fenice score was devised using an objective consequence of crowding, i.e., the increase in waiting time for patients triaged with deferrable urgency, we relied on the staff perception for its assessment. In this context, we involved multiple operators from a wide range of professional roles, to mitigate concerns about subjectivity and ensure a collective perspective. Formally, we investigated what is referred to as construct validity, which refers to how well a tool actually measures the theoretical scheme, or construct, it was designed to measure [17]. This can be done by comparing the indicator of interest against another indicator measuring the same, or similar, aspects of the construct [18].

Overall, we found both scores to be strongly associated with staff perceptions of crowding, with the Fenice score showing slightly higher levels of agreement, even though the difference between scores was marginally non-significant, and a greater ability to explain variability in these perceptions, particularly when stratified by professional role. These findings are notable given that the Fenice crowding score is simple and fully automatable, relying solely on the total number of patients in the ED at any given time.

This study demonstrated the feasibility of collecting real-time staff perception of crowding via SMS-based questionnaires. While the overall response rate of 13.7% may seem modest, it should be noted that the probability of the text message reaching staff during their shift in the ED was only 21.9%. Consequently, the 13.7% response rate reflects a participation rate of 62.6% among potential respondents, which is higher than the average response rate (52.7%) for studies that utilized data collected from individuals [19], and is in line with what is considered acceptable (> 60%) for survey research [20]. It is also noteworthy that responses were provided consistently over a 2-month period, with most replies received within an hour of message delivery. The heterogeneity in response rates across roles suggests that engagement varied by position: the chief nurse and ED director responded most frequently, while other roles had lower participation rates. This discrepancy may reflect differences in workload, accessibility to mobile devices during shifts, or the perceived relevance of the survey. Future studies could explore strategies to improve participation.

Our study confirms substantial heterogeneity in perceptions of crowding among different professional roles. Healthcare assistants and boarding nurses perceived the ED as more crowded than their colleagues did, while process management nurses tended to perceive it as less crowded. This may be due to differences in daily tasks, exposure to specific workflow bottlenecks, or responsibility for managing patient flow.

Importantly, although perceived crowding and workload pressure were correlated, they were not interchangeable concepts. In this regard, it is possible that the similarity of the scales on which the two concepts were collected generated a carryover bias, whereby a respondent’s answer to a question is influenced by the response given to a previous question. In future, to avoid spurious correlation and to estimate the threshold of ED crowding levels triggering a marked increase in the pressure of the different professional roles in the ED, similar studies should collect data on workload pressure using a dichotomous answer. Nonetheless, despite the similarity of the overall scores for these two questions, we observed statistically significant differences across roles, suggesting that workload and task-specific stressors influence perceptions of crowding and feeling of being rushed in different ways. This distinction highlights the importance of treating these two aspects as complementary indicators of ED pressure.

The strong correlation observed between the Fenice crowding score and staff perceptions (*ρ* = 0.709) highlights its potential as a valid real-time crowding proxy, even considering that this correlation was found to be almost significantly better than the more complex NEDOCS (*ρ* = 0.689; *p* = 0.06). The slightly higher explanatory power of the Fenice crowding score in regression models (*R*^2^ = 0.65 vs. 0.59) suggests a better alignment with staff experience. This result is particularly relevant because the two scores were constructed on very different premises. The NEDOCS was specifically developed to predict how operators perceive crowding and, therefore, should be considered as a subjective crowding score. In contrast, the Fenice indicator was developed to measure a quantifiable consequence of overcrowding, i.e., longer waiting times for patients with minor or deferrable urgency. It is therefore an objective crowding score. Interestingly, the objective crowding score performed equally well, if not better, than the subjective one, even when the two are evaluated using the same parameter that was used to develop the subjective score.

Both scores were moderately correlated with the feeling of being rushed. The values of the correlation coefficients below 0.60 highlight the limitations of crowding indices in capturing work-related stress and pace. These findings reinforce the idea that indicators of crowding should not be interpreted in isolation but, rather, in conjunction with other measures that capture the human impact of pressure in EDs.

Another notable contribution of this study is the exploration of role-specific responses to crowding indicators. Using GAM, we found that while most roles showed a linear relationship between the Fenice crowding score and perceived crowding, some roles, particularly those of emergency room nurses, displayed stepwise trends. This may reflect a threshold-based perception, whereby staff experience relatively manageable conditions until a tipping point is reached, after which perceived crowding escalates sharply. Such non-linearities could help categorize the indicator into meaningful classes for the operators, rather than arbitrary ones.

Interestingly, while the NEDOCS and the Fenice scores were strongly correlated in our single-center study (*ρ* = 0.848), this association was not observed in a broader group of EDs across Lombardy, where *ρ* values ranged from 0.238 to 0.992. As a result, assessments of ED crowding in Lombardy varied considerably depending on the indicator used, as reflected by differing rankings of crowding levels. This suggests that the two scores may diverge more substantially depending on the context, particularly where differences in hospital organization, patient flow, or ED size affect the calculation and interpretation of NEDOCS. The study site, being a large university hospital with established digital monitoring systems, may have inherently favored a closer correlation between the two scores. Alternatively, this result could reflect the specific approximations used by the study ED for the NEDOCS components that cannot be automatically calculated from electronic clinical records. Such alignment may not apply to settings with less structured or more variable data collection systems, which highlights one of the major limitations of the NEDOCS when applied outside of its original development context [10]. Conversely, the Fenice score only relies on the availability of the overall number of patients present in the ED. Since this information is unambiguous and can be easily retrieved from any ED software at any given time, the Fenice score can be easily used for real-time monitoring.

A major strength of this study is its real-time, repeated-measures design, leveraging modern, low-burden digital tools for data collection. This ensured frequent sampling, minimizing recall bias, and allowing for dynamic assessment of crowding perception. Additionally, the blinding of staff to crowding scores prevented response bias. Finally, the inclusion of all ED professional roles allowed for a nuanced interpretation of how differently staff members experience crowding.

The main limitation of the study consists in its monocentric nature and the relatively short observation period during winter, a time of year that is often associated with high ED activity. The generalizability of the results to other settings, seasons, or hospitals of different sizes and organizational structures remains to be verified. For example, the ED size may influence the agreement between staff perception and the crowding score, as healthcare professionals in larger departments with multiple areas or workstreams may have a partial view of overall crowding. Agreement may also vary with patient case mix, as the considered crowding scores do not account for the reason for ED arrival or clinical presentation; thus, the disproportionate impact of patients posing a substantial care burden may not be fully captured by the scores. Finally, our findings may not immediately extend to lower-demand periods since perceptions of crowding under average conditions may differ depending on whether they occur during times of unusually high or low pressure. Moreover, seasonal fluctuations in disease patterns, such as influenza or acute coronary syndromes, can alter the case mix of ED presentations and, in turn, affect the agreement between the Fenice score and staff perceptions of crowding. Future research should address these issues in broad multicenter studies.

Other limitations must be acknowledged. First, staff perceptions were collected at two fixed times of day, every day. The generalizability to other timing should be consequently assessed. Second, the voluntary nature of participation may have introduced a selection bias, with individuals more sensitive to ED crowding potentially being more likely to respond. Unfortunately, we did not track responses from the same individuals, thus we cannot evaluate whether the results differ across subjects with different response rates. Third, although we analyzed over 800 on-duty responses, the lack of role data in 31.6% of the responses limited our ability to perform fully stratified analyses. Finally, while the Fenice crowding score performed slightly better than the NEDOCS in some comparisons, this difference was not statistically significant, so the results should be interpreted with caution.

In conclusion, this study demonstrates that assessing ED staff perceptions of crowding in real time is feasible and informative. Both NEDOCS and the newly proposed Fenice crowding indicator correlate strongly with these perceptions. While the simplicity of the Fenice score and the possibility to fully automate its calculation make the score operationally appealing, further studies specifically designed to validate it across different settings are warranted.

## Supplementary Information

Below is the link to the electronic supplementary material.

Supplementary file1 (DOCX 415 KB)

## Data Availability

Data analyzed in the study might be shared on request.
