# Supplementary material for: Measuring emergency department crowding: comparing the NEDOCS and Fenice crowding scores against staff perception
Source: Intern Emerg Med. 2025 Dec 12;21(8):3055–63. doi: 10.1007/s11739-025-04216-7 (PMC13751802; doi:10.1007/s11739-025-04216-7)
Supplement: Supplementary file 1 [file 11739_2025_4216_MOESM1_ESM.docx]

# Supplementary materials of

# “Measuring Emergency Department crowding: comparing the NEDOCS and Fenice crowding scores against staff perception”

## S1. Supplementary methods

### S1.1 Calculation of the Fenice ED-crowding score

We applied the methodology described by Signorini et al. (2024) to compute the Fenice ED-crowding score on the study ED. We used the 2023 data of ED accesses and patients’ waiting time to evaluate the relationship between the total number of patients in the ED and the waiting time for the first clinical assessment, stratified by triage code. Importantly, the Fenice ED-crowding score was originally developed using data from EDs where a 4-level triage system was in place. In particular, the score was proposed based on the relationship observed for patients with minor or deferrable urgency, who were assigned the same triage level. In contrast, a 5-level triage was in place in the study ED and, specifically, deferrable and minor urgency patients were assigned different triage codes. The figure below shows the estimated lines for the five triage codes and the related confidence bands.

The steepest line was the one of patients with deferrable urgency (blue code). This suggests that this is the group of patients for whom the waiting time is most sensitive to the rising patient volumes in the ED. Thus, the calculation of the score was based on the waiting time of patients with deferrable urgency. The Fenice ED-crowding score can be easily calculated by inputting the total number of patients present in the ED in the equation represented by the regression line for deferrable urgency patients.

In particular, we studied the Fenice ED-crowding score as a quantitative, continuous indicator, expressed as the estimated average waiting time (in minutes) based on the number of patients present in the ED. In our previous work, we proposed a strategy to categorize the crowding level in discrete levels (Signorini et al., 2024). This can be done by defining thresholds in waiting times (for example, 15, 30, 60, 90, 150, and 210 minutes) and, through the estimated line, computing the cutoffs on the number of patients present in the ED corresponding to these thresholds. The figure below represents the estimated line for deferrable urgency patients along with the threshold values on waiting time and the corresponding cutoffs in the number of patients.

The cutoffs define an objective crowding indicator and its interpretation is consistent across all EDs. At the lowest crowding level, patients with deferrable urgency wait less than 15 minutes on average. In the study ED, the lowest level is attained when less than 11 patients are present in the ED. In contrast, at the highest crowding level, the expected waiting time for deferrable urgency patients exceeds 3.5 hours for all EDs. In this ED, this level is attained when more than 52 patients are present in the ED.

In line with the calculation of the Fenice indicator in the study ED, we estimated the score for the 83 general EDs in the Lombardy Region during the study period. Notably, the triage system transitioned from four to five levels in 2023. As a result, we re-assessed the linear relationship between the number of patients present in the ED and their waiting time for the first clinical assessment, stratified by triage code. Consistently with the analysis and results in the study ED, we decided to focus on patients with deferrable urgency.

The Fenice crowding score was defined using data from the last six months of 2024 for all 83 EDs. The centre-specific linear relationship for deferrable-urgency patients was estimated and applied to the hourly number of patients present in the EDs. Notably, the crowding score has the same interpretation for all EDs, i.e., the average waiting time of deferrable-urgency patients. As a result, although the relationship is specific to each centre, the resulting Fenice score allows for direct comparison of crowding levels across the Lombardy EDs and the ED examined in this study.

### S1.2 Calculation of the NEDOCS score

In the study ED, the NEDOCS is computed using the following variables:

A) Number of ED beds (stretcher included).
B) Number of hospital beds.
C) Number of patients present in the ED.
D) Number of ventilated patients. Because this number cannot be extracted from electronic health records automatically, at any given time it is approximated using the number of patients triaged as immediate emergencies (red code) within the past hours.
E) Maximum hospitalization time (waiting time of patients requiring an hospital bed – hours).
F) Number of patients waiting for hospitalization.
G) Maximum admission time (waiting time of patients for the first physician assessment – hours).

The NEDOCS score is calculated according to the formula:

$$NEDOCS=85.8\cdot\frac{C}{A}+600\cdot\frac{F}{B}+13.4\cdot D+0.93\cdot E+5.64\cdot G-20$$

The NEDOCS crowding level is classified following the classification reported in the table below.

| Indicator level | NEDOCS cutoff | Crowding level |
| --- | --- | --- |
| 1 | 0-50 | Normal – green |
| 2 | 51-100 | Busy – yellow |
| 3 | 101-140 | Overcrowded – orange |
| 4 | 141-180 | Severe – red |
| 5 | >180 | Disaster – black |

## S2. Supplementary figures

**Figure S1.** Number of received responses per day during the study period.

**Figure S2.** Time of reception of responses.

**Figure S3.** Comparison of the Spearman correlation coefficients of the Fenice crowding score and NEDOCS in the study ED (red bar) and all general Lombardy EDs (average correlation in blue).
